# Lifetime prevalence of novel psychoactive substances use among adults in the USA: Sociodemographic, mental health and illicit drug use correlates. Evidence from a population-based survey 2007–2014

**DOI:** 10.1371/journal.pone.0241056

**Published:** 2020-10-30

**Authors:** Jessica Neicun, Justin Christopher Yang, Hueyjong Shih, Pranay Nadella, Robin van Kessel, Attilio Negri, Kasia Czabanowska, Carol Brayne, Andres Roman-Urrestarazu

**Affiliations:** 1 Department of International Health, Care and Public Health Research Institute (CAPHRI), Faculty of Health, Medicine and Life Sciences, Maastricht University, Maastricht, The Netherlands; 2 Institute of Public Health, University of Cambridge, Cambridge, United Kingdom; 3 Novel Psychoactive Substance Unit, Centre for Clinical & Health Research Services, University of Hertfordshire, Hatfield, United Kingdom; 4 National Institute of Public Health, Warsaw, Poland; Chiang Mai University Faculty of Medicine, THAILAND

## Abstract

**Introduction:**

As Novel psychoactive substances (NPS) are conceived to mimic the effects of common illicit drugs, they represent a serious public health challenge due to the spike in intoxications and fatalities that have been linked to their use. This study aims to provide epidemiological data on NPS use in the USA, determining lifetime prevalence of use and defining demographic, socioeconomic, drug use patterns and mental health correlates.

**Methods:**

This study uses secondary data from the US National Survey on Drug Use and Health (NSDUH), which is a large cross-sectional population-based survey carried out annually in the USA. We analysed data from 2007–14 (N = 307,935) using bivariate descriptive analysis and binary logistic regression to calculate prevalence and determine factors underlying NPS consumption. Adjusted odds ratios (OR) with 95% CI’s were calculated for a set of selected independent variables.

**Results and discussion:**

Our analysis NSDUH from 2007–14 highlights an increase in NPS use among adults, especially among white young men aged 18 to 25. Although the level of education of NPS users was relatively higher as compared to non-users, NPS users seemed to have a less wealthy situation. However, socioeconomic vulnerability appeared to be less important than mental health issues as a correlate to NPS use. NPS users seem to have followed a pattern of polysubstance use throughout their life, which involves both traditional illicit drugs and classic synthetic drugs. As NPS use seemed to be more prevalent among people having mental health issues, the rise in their use may have a negative impact on population mental health outcomes.

**Conclusion:**

Further comparative research on trends in NPS use and potential public health responses would be instrumental for developing appropriate health interventions, including drug checking, education for users and training for healthcare professionals working both within emergency wards and in/outpatient addiction and mental health services.

## 1. Introduction

Over the last few decades, the emergence of Novel Psychoactive Substance (NPS), sometimes also called “legal highs”, “research chemicals” or “designer drugs”, has become a topic of interest for researchers, policy officers, healthcare providers and enforcement agencies in high-income countries [1–9]. NPS are conceived to mimic the pharmacological effects of common illicit drugs. As a result, a variety of uncontrolled new substances is regularly appearing in the international drug market to circumvent current drug legislation by continuously changing their chemical composition [10–14]. The number of NPS identified by authorities worldwide has increased by more than 400% since 2009. Globally, 950 new substances have been detected by the end of 2019 (UNODC Early Warning Advisory), of which 790 are already available in the European market according to the EU Early Warning System [15, 16]. NPS represent a serious public health challenge due to the lack of awareness of their actual composition, as well as their potency, effects and risk profile. NPS stimulants and novel synthetic opioids–most of which are much stronger than morphine–have already been linked to a spike in intoxications and fatalities in the USA and Europe [17–22]. Although the available epidemiological evidence on the use of NPS has increased in recent years, knowledge of lifetime prevalence rates is limited due to the scarcity of relevant population-based surveys with adequate sampling design [23–39]. We aim to address this gap by putting forth a population-based study assessing lifetime prevalence of NPS use among adults in the USA. Our study utilised secondary data from the U.S. National Survey on Drug Use and Health (NSDUH), which collects information on alcohol, tobacco and illegal substance use, misuse of prescription medicines and mental health. We examined lifetime rates of NPS use between 2007 and 2014, with the aim of evaluating (1) the socio-demographics characteristics of NPS users, as well as (2) the possible association between illicit drug use patterns, the (3) mental health needs of this population and (4) the patterns of NPS use and substances preferred by users.

## 2. Methods

### 2.1 Sample and procedures

This study analyses secondary data from the NSDUH. It is therefore no primary recruitment of participants since all data has already been collected nor it is possible to obtain consent from participants included in this study. Data for adults aged 18 and over were included from the 2007–14 cohorts of the NSDUH, an annual, cross-sectional population-based survey of civilian, non-institutionalised individuals (*N* = 307,935). This eight-year timeframe was chosen due to notable changes made to the survey questionnaire and data collection procedures in 2015, affecting comparability over time [40]. The sample strategy for this period consists of a multistage, deeply stratified area probability sample design. First, census tracts are defined using equally sized geographical partitions of the 50 U.S. States plus de District of Columbia (first stage). These areas are then partitioned into smaller geographic areas or “segments” (second stage). Afterwards, a number of dwelling units are selected according to a selection rate based on a state’s population size (third stage). Finally, individuals (fourth stage) are selected within dwelling units based on five age-group classifications: 12 to 17, 18 to 25, 26 to 34, 35 to 49, and 50 or older. The survey is administered *via* face-to-face interviews using computer-assisted interviewing (CAI) and audio computer-assisted self-interviewing (ACASI) to increase respondents’ cooperation and willingness to report honestly about topics such as illicit drug use behaviour and mental health issues. Questions pertaining to the use of regulated substances are self-administered through ACASI [41].

### 2.2 Demographics

We categorized individuals from the NSDUH according to the following age groups: 18–25, 26–34, 35–49 and 50 years or older. Sex was coded as either Male or Female. Respondent ethnic background was coded as White, African American, Hispanic and Other (including Native American and Asian or Pacific Islander). Education was coded along the following categories: Elementary (7th grade or less), Secondary (8th to 12th grade), and Post-Secondary (13^th^ grade/Freshman or higher). Marital status was coded as either Single–including widowed, divorced and separated–or Married. Metropolitan size was coded as Large, Small or Non-metropolitan based on 2010 census data and 2009 Core Based Statistical Area classifications provided by the Office of Management and Budget (OMB) [41].

### 2.3 Socioeconomic variables

Employment status was categorized as Full-time, Part-time, Not employed (including unemployed and not in labour force). Household annual income was categorised as: less than $20,000; $20,000 - $49,999; $50,000 - $74,999; $75,000 or more. A dichotomous variable was created to indicate whether a respondent received any form of government assistance (i.e. supplemental security income, food stamps, cash assistance, and/or non-cash assistance). The respondent’s primary health insurance was coded as Private, Medicare (covering people aged 65 and older, as well as people with disabilities or chronic health conditions), Medicaid (for individuals whose resources are insufficient to pay for healthcare regardless of their age), Tricare & Veterans Administration (VA) (for military veterans), other, or Uninsured. Additionally, based on the four indicators described above, an aggregate index of socioeconomic vulnerability was created by summing the number of vulnerability attributes observed (i.e. no employment, annual income lower than $20,000, no health insurance or eligible for Mediaid, receiving government assistance). The index values range from 0 (which indicates no vulnerability attributes) to high (when at least 3 vulnerability attributes were observed).

### 2.4 Use of NPS

We considered lifetime use of 64 NPS as our outcome variable. These substances were spontaneously mentioned by respondents through open-ended questions about the recreational use of (1) prescription drugs (classified by the survey into four categories: pain killers, sedatives, stimulants and tranquilizers), (2) hallucinogens (other than LSD, PCP, peyote, mescaline, psilocybin and ecstasy), (3) inhalants, (4) non-prescription cough or cold medicines and (5) special drugs such as injectable stimulants. For the purpose of this study, we defined as NPS according to two criteria: 1) their international legal status (substances not classified under Schedules I and II of the 1971 UN Convention on Psychotropic Substances); 2) their recreational use among general population, defined by the questions about their consumption for the experience or feeling they cause (“for kicks or to get high”). A few exceptions to these rules were made with controlled substances whose recreational use has remained limited until recently (e.g. Bromo mescaline, Brolamfetamine (DOB), MDA, PMA) (S1 Table).

We listed 63 reported substances and classified them into four NPS categories according to their pharmacological effects: (1) hallucinogens, (2) stimulants, (3) depressants and (4) synthetic cannabinoids. Among the 64 substances reviewed, we identified 29 phenethylamines (22 hallucinogens and 7 stimulants), 17 tryptamines, 5 synthetic cathinones (including 1 generic category encompassing three different street names), 3 synthetic cannabinoids (including 1 generic category compassing nine different street names), 2 ergolines, 2 dissociatives, 2 benzodiazepines, 1 aminoindane, 1 piperazine, 1 inhalant and 1 synthetic opioid (S1 Table). We subsequently created dichotomous variables indicating lifetime use of at least one substance of each category. Finally, an aggregate indicator for lifetime NPS use was created based on the reported use of at least one of the four categories.

### 2.5 Use of traditional illicit drugs and “classic synthetic drugs”

In order to better characterise the drug use patterns of NPS users, two aggregate dichotomous variables were created. Similar to the construction of the lifetime NPS use variable, we created an aggregate indicator for lifetime use of illicit drugs based on the use of at least one of the four traditional illicit drugs included in the NSDUH. This aggregate indicator was constructed based on the data collected through the NSDUH core questions on lifetime use of cocaine, crack, heroin and cannabis. Additionally, an aggregate indicator for lifetime use of “classic synthetic drugs” was also constructed based on the data from NSDUH core questions on lifetime use of LSD, ecstasy and PCP, as well as the use of so-called (2) “special drugs” such as methamphetamine and GHB, as defined by NSDUH [42]. For the purpose of this study, “classic synthetic drugs” were defined as commonly used synthetic psychoactive substances already placed under international control and whose chemical structures, pharmacology and effects are well known by the scientific community.

### 2.6 Past-year mental health

Past-year prevalence of four mental health indicators were included in the study. First, we used the Serious Psychological Distress variable (SPD), which is based on the Psychological Distress Scale (K6) that gathers information regarding how frequently individuals experience symptoms of psychological distress over a time period (e.g. over the past month or over the worst month of the past year that was not the past 30 days), with values ranging from 0 to 24. Symptoms of distress include feeling nervous, feeling hopeless, feeling restless or fidgety, feeling so sad or depressed that nothing could cheer one up, feeling that everything was an effort, and feeling down on oneself, no good, or worthless. For each of these six items, responses of "all of the time" were coded 4, "most of the time" were coded 3, "some of the time" were coded 2, "a little of the time" were coded 1, and "none of the time" and all other responses were coded 0. These assigned values were summed across the six items to calculate a past month K6 total score and a K6 score for the worst month of the past year other than the past 30 days. Respondents were classified as having had a past year SPD if their K6 score was 13 or higher (based on the higher score between the past month K6 total score and the K6 score in the worst month of the past year other than the past 30 days). Conversely, they are classified as not having had a past year SPD if their K6 score was lower than 13 [42].

Second, we used the past-year Major Depressive Episode (MDE) indicator. Respondents were defined as having MDE in the past year if they had a lifetime MDE and a period of time in the past 12 months when they felt depressed or lost interest or pleasure in daily activities for 2 weeks or longer, while also having some of the other symptoms for lifetime MDE. Respondents were defined as having had a lifetime MDE if they had five or more of nine symptoms for MDE in the same 2-week period in their lifetime, in which at least one of the symptoms was a depressed mood or loss of interest or pleasure in daily activities. Symptoms for MDE include: (1) depressed mood most of the day; 2) markedly diminished interest or pleasure in all or almost all activities most of the day; 3) changes in appetite and weight; 4) insomnia or hypersomnia; 5) psychomotor agitation or retardation; 6) fatigue or loss of energy; 7) feelings of worthlessness; 8) diminished ability to think or concentrate or indecisiveness; 9) recurrent thoughts of death or recurrent suicide idea. Respondents with no lifetime MDE or respondents with lifetime MDE but no period of depression lasting 2 weeks or longer while having other symptoms were defined as not having past year MDE. It should be noted at this point that the NSDUH makes no exclusions for MDE caused by medical illness, bereavement, or substance use disorders [42].

Third, we created an indicator of past-year access to mental health treatment that encompasses access to in/outpatient mental health treatment and use of prescription medicine for a mental health condition [42]. Finally, we use the indicator of "unmet mental health need" defined by NDSUH as having perceived a need for mental health treatment/counselling that was not received during the past 12 months [42]. Respondents could agree with multiple diagnostic questions, so these indicators are not mutually exclusive. Reasons for not receiving mental health treatment/counselling that was needed in the past year were asked by the NDSUH through a set of questions, yet they were not included in this study.

### 2.7 Imputed data

Since 1999, the NSDUH addresses item nonresponse using an imputation method known as predictive mean neighbourhood (PMN), which is applied in a stepwise fashion: (1) response propensity adjustment; (2) prediction modelling; and (3) hot-deck imputation [41]. Data imputation has been extensively used for variables having a larger proportion of missing values (e.g. ethnicity and government assistance) and slightly less for variables such as education, marital status, income, and health insurer [41].

### 2.8 Statistical analyses

All statistical analyses were performed in Stata 14.2. NSDUH datasets from individual years were combined into a single file for analysis to allow for year to year comparisons. In order to estimate the annual average of the number of individuals who engaged in a particular behaviour based upon pooled data from 2007–14, adjusted analysis weights were created by dividing annual weights by eight (the number of cohorts under study) according to the NSDUH Public Use File Codebook [42]. Thus, all analyses used the analytical weights provided with the datasets to account for the complex survey design of the NSDUH. We utilised a bivariate descriptive analysis with weighted least squares to investigate how demographics, socioeconomic characteristics, drug use patterns and mental health were related to NPS use. For the multiple response NPS variable, the significance of the differences among the proportions of NPS categories was evaluated through the non-parametric Cochran’s Q test, which yielded highly significant differences in the proportions of the four NPS categories (Cochran's chi2(3) = 1133.129, p<0.01) [43]. In order to identify which factors were more related to NPS use, we performed a pairwise correlation at *p*<0.05; yet it appeared that none of the factors had a strong correlation with the outcome variable. As a result, we performed a binary logistic regression for NPS use and a set of independent variables selected upon our previous knowledge of the topic. Adjusted odds ratios (OR) with 95% CI’s were calculated for the selection of the independent variables included in the model (age, sex, marital status, ethnicity, education, past-year SPD, MDE, unmet mental health need, access to mental health treatment, lifetime use of traditional illicit drugs and classic synthetic drugs). As the available epidemiological evidence on the use of NPS is still scarce, one of our objectives was to provide relevant population-based data. Hence, we decided to use adjusted odds ratios (OR) as they are commonly used in epidemiology to compare the relative odds of the occurrence of a particular outcome (e.g. NPS use), given exposure to a series of variables of interest (e.g. sociodemographics, mental health characteristic, drug use patterns). Although, the 95% confidence interval (CI), used to estimate the precision of the OR, may not be the most adequate measure of accuracy for the purpose of this study.

## 3. Results

### 3.1 Variations in NPS use over time

**Fig 1:**
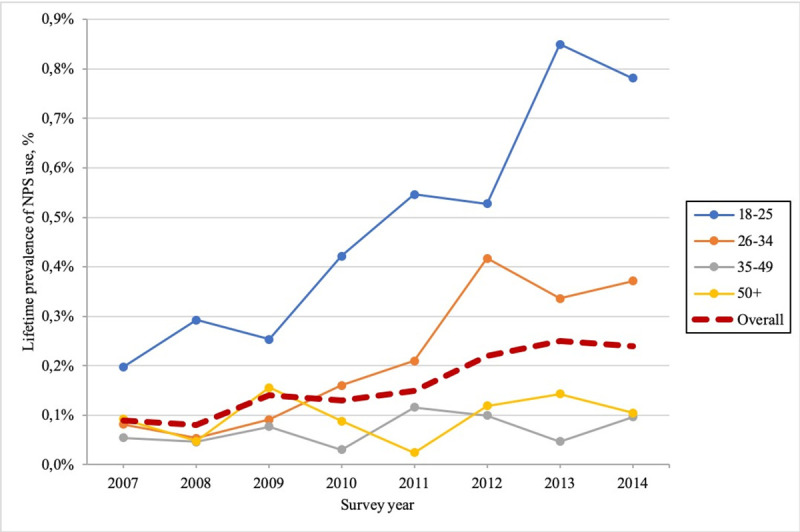
Lifetime prevalence of NPS use by age group, 2007–14.

Consumption of NPS showed an 167% increase between 2007–14. In 2007, the estimate for lifetime NPS use among adults was 0.09% (95%; CI = 0.06%-0.16%), while 0.24% (95%; CI = 0.19%-0.31%) of adults had used an NPS seven years later (Fig 1).

The increase in NPS use was notably led by young people–particularly by those between the ages of 18 and 25 –whose lifetime prevalence of NPS use exhibited a fourfold increase between 2007–14. The exception to this upward trend was the pattern followed by people between aged 50 or older, whose prevalence of NPS use remained stable at around 0.1% during the same period (Fig 1).

**Fig 2:**
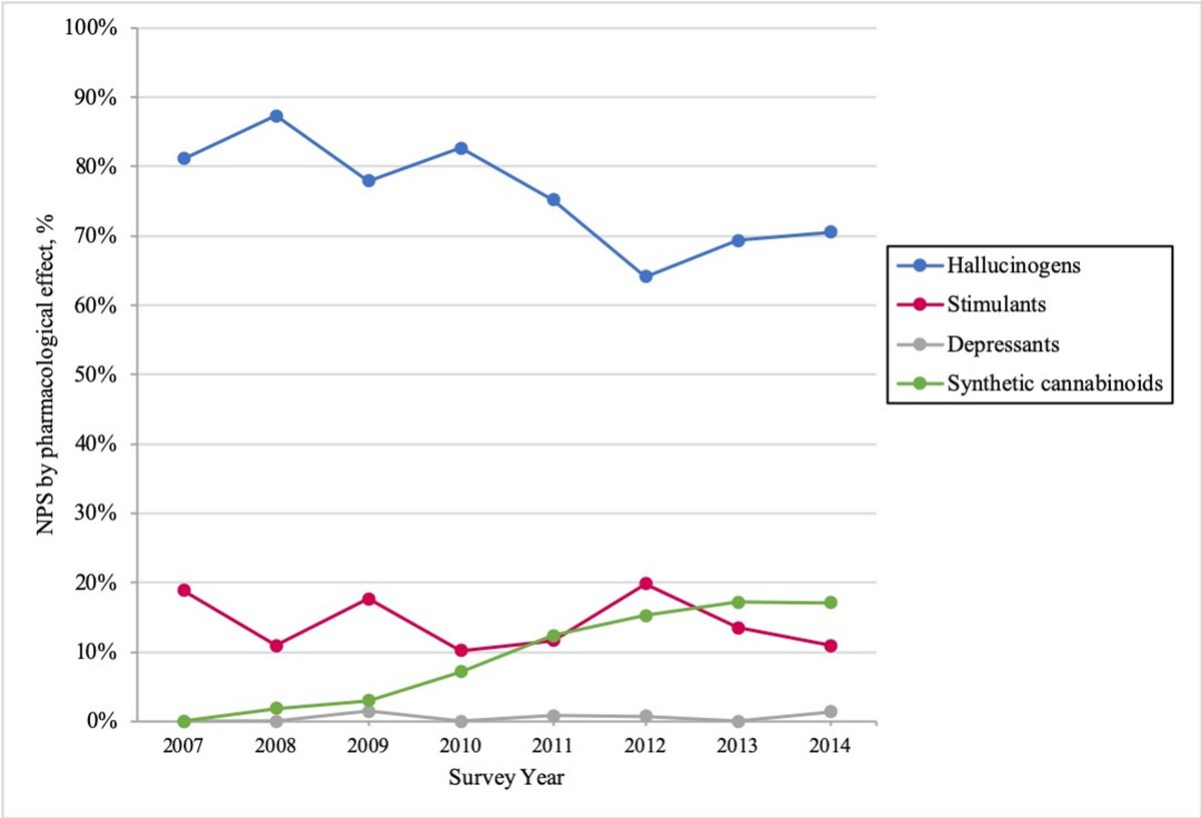
Evolution of NPS reported use by pharmacological effect, 2007–14.

Regarding the evolution over time by NPS category, it is worth to note that the use of synthetic cannabinoids rose steadily between 2007–2014: it accounted for 0.8% of reports in 2008 (when it was first mentioned by respondents), reaching up to 17.1% in 2014. On the other hand, the use of hallucinogens and stimulants showed a downward trend during the same period (-11% and -8% of reports respectively). Meanwhile, the use of depressants remained stable at around 1% of reports (Fig 2).

### 3.2 Lifetime use of NPS by drug class

**Fig 3:**
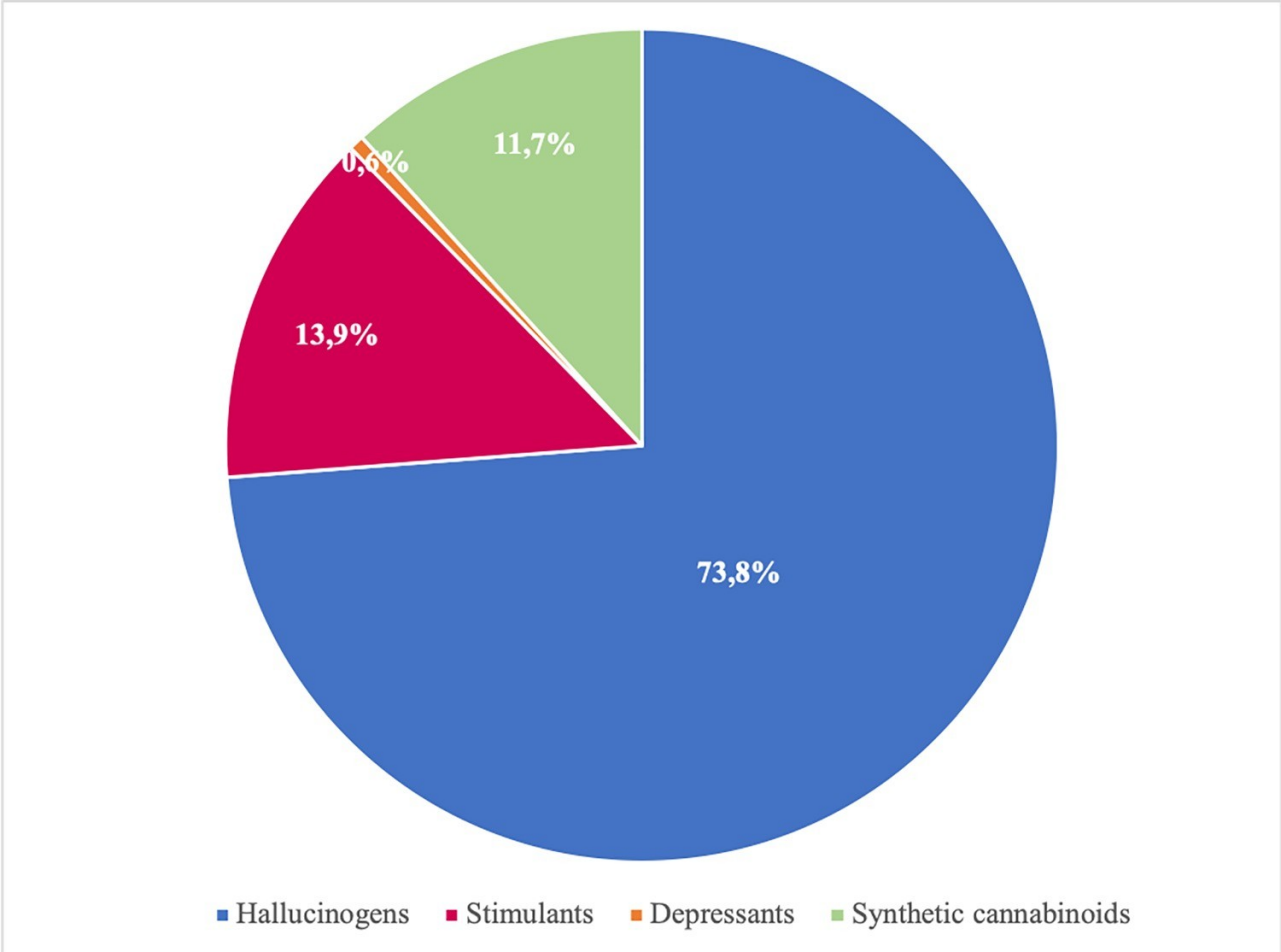
Proportion of NPS reported use, by pharmacological effect, 2007–14.

Measures of lifetime NPS use were obtained for four NPS categories. The differences in the proportions of these categories were highly significant, with hallucinogens being the most frequently reported by NPS users (73.8%), followed by stimulants (13.9%) and synthetic cannabinoids (11.7%). The use of depressants (0.6%) appeared to be less widespread among our study population (Fig 3). The overall estimate of lifetime NPS use for the seven-year period was 0.17% (95% CI = 0.15%-0.19%), which corresponds to 385 thousand NPS users (Table 1).

**Table 1:** Lifetime prevalence rate of NPS use among adults by sociodemographic variables, characteristics of Users and Non-users of NPS, 2007–14.

	Lifetime Prevalence Rate		NPS Users		Non-users of NPS	
	%	95% CI	%	95% CI	%	95% CI
Overall estimate	0.17	(0.15–0.19)				
Unweighted sample			n = 837		n = 307 148	
Weighted sample			n = 385 011		n = 230 802 805	
**Age**						
18–25	0.49	(0.43–0.55)	43.12	(37.54–48.87)	14.66	(14.45–14.87)
26–34	0.22	(017–0.28)	20.64	(16.44–25.58)	15.79	(15.58–16.01)
35–49	0.07	(0.05–0.10)	11.43	(7.95–16.16)	27.05	(26.78–27.32)
50 and older	0.1	(0.07–0.14)	24.82	(18.27–32.76)	42.5	(42.08–42.92)
**Sex**						
Male	0.27	(0.23–0.31)	77.34	(71.94–81.96)	48.17	(47.87–48.46)
Female	0.07	(0.06–0.09)	22.66	(18.04–28.06)	51.83	(51.54–52.13)
**Ethnicity**						
White	0.21	(0.19–0.24)	84.54	(77.75–89.54)	67.25	(66.86–67.63)
African American	0.04	(0.02–0.08)	2.67	(1.35–5.20)	11.59	(11.31–11.88)
Hispanic	0.08	(0.06–0.11)	7.25	(5.33–9.78)	14.3	(14.03–14.57)
Other	0.13	(0.05–0.35)	5.54	(2.14–13.59)	6.86	(6.66–7.07)
**Marital Status**						
Single	0.28	(0.25–0.31)	77.21	(70.27–82.93)	46.58	(46.22–46.94)
Married	0.07	(0.05–0.10)	22.79	(17.07–29.73)	53.42	(53.06–53.78)
**Metropolitan Area Size**						
Non-Metropolitan	0.13	(0.08–0.21)	12.83	(8.28–19.35)	16.15	(15.79–16.52)
Small Metropolitan	0.18	(0.15–0.21)	32.52	(28.19–37.18)	30.25	(29.69–30.83)
Large Metropolitan	0.17	(0.14–0.20)	54.65	(48.14–61.01)	53.6	(53.10–54.09)
**Education**						
Elementary	0.02	(0.01–0.06)	0.04	(0.14–1.14)	3.29	(3.15–3.43)
Secondary	0.12	(0.11–0.14)	31.11	(26.99–35.55)	41.52	(41.19–41.85)
Post-secondary	0.21	(0.18–0.24)	68.49	(64.01–72.65)	55.19	(54.84–55.54)
**Employment**						
Full-Time	0.16	(0.14–0.19)	49.94	(44.37–55.51)	51.24	(50.94–51.55)
Part-Time	0.3	(0.23–0.40)	25.29	(19.94–31.50)	13.87	(13.67–14.07)
Not employed	0.12	(0.09–0.15)	24.78	(19.39–31.08)	34.89	(34.57–35.22)
**Income**						
Less than 20,000	0.29	(0.24–0.37)	32.4	(26.83–38.52)	18.29	(17.98–18.61)
20,000 to 49,999	0.13	(0.11–0.17)	26.3	(21.31–31.98)	32.48	(32.18–32.77)
50,000 to 74,999	0.14	(0.10–0.20)	14.89	(10.88–20.04)	17.28	(16.98–17.59)
More than 75,000	0.14	(0.11–0.17)	26.41	(21.61–31.85)	31.95	(31.49–32.42)
**Health Insurer**						
Private	0.15	(0.13–0.17)	59.65	(52.95–66.01)	66.68	(66.29–67.08)
Medicare	0.18	(0.08–0.42)	8.62	(3.73–18.66)	7.84	(7.63–8.06)
Medicaid	0.13	(0.09–0.18)	4.96	(3.50–7.00)	6.46	(6.31–6.60)
Tricare & VA	0.13	(0.06–0.31)	1.41	(0.61–3.21)	1.75	(1.67–1.84)
Other	0.37	(0.21–0.67)	4.12	(2.32–7.21)	1.84	(1.77–1.92)
Uninsured	0.23	(0.18–0.29)	21.23	(16.64–26.68)	15.43	(15.21–15.64)
**Receives Government Assistance**	0.19	(0.16–0.24)	20.73	(16.64–22.52)	17.84	(17.60–18.09)
**Economic Vulnerability**						
No vulnerability	0.14	(0.11–0.17)	37.48	(31.41–43.97)	45.51	(45.16–45.86)
Low (1 attribute)	0.2	(0.15–0.25)	36.29	(28.19–43.10)	29.87	(29.53–30.22)
Moderate (2 attributes)	0.19	(0.14–0.25)	15.79	(11.73–20.91)	13.95	(13.71–14.19)
High (3 or 4 attributes)	0.18	(0.14–0.23)	11.44	(8.51–15.21)	10.67	(10.44–10.91)
**Past-Year Serious Psychological Distress**						
No						
Yes	0.42	(0.33–0.52)	26.38	(22.64–27.61)	10.51	(10.35–10.68)
**Past-Year Major Depressive Episode**						
No						
Yes	0.45	(0.34–0.58)	18.14	(14.69–19.50)	6.73	(6.60–6.86)
**Past-Year Perceived Unmet Mental Health Need**						
No						
Yes	0.51	(0.39–0.65)	15.03	(12.76–16.90)	4.91	(4.80–5.02)
**Past-Year Received Mental Treatment**						
No						
Yes	0.32	(0.25–0.41)	26.93	(24.80–32.82)	14.01	(13.80–14.22)
**Lifetime use of traditional illicit drugs**						
No use of traditional illicit drugs						
Any traditional illicit drug	0.36	(0.32–0.40)	98.71	(97.38–99.37)	45.41	(45.10–45.73)
*Cocaine*	*0*.*88*	*(0*.*78–1*.*00)*	*85*.*46*	*(82*.*43–88*.*05)*	*15*.*97*	*(15*.*77–16*.*18)*
*Crack*	*1*.*28*	*(1*.*04–1*.*57)*	*29*.*19*	*(24*.*22–34*.*70)*	*3*.*76*	*(3*.*65–3*.*87)*
*Heroin*	*2*.*92*	*(2*.*29–3*.*73)*	*32*.*08*	*(26*.*04–38*.*79)*	*1*.*78*	*(1*.*69–1*.*87)*
*Cannabis*	*0*.*36*	*(0*.*33–0*.*41)*	*98*.*66*	*(97*.*31–99*.*34)*	*44*.*99*	*(44*.*67–45*.*30)*
**Lifetime use of classic synthetic drugs**						
No use of synthetic drugs						
Any classic synthetic drug	1.09	(0.97–1.22)	92.43	(89.56–94.56)	14.02	(13.86–14.18)
*LSD*	*1*.*35*	*(1*.*19–1*.*52)*	*82*.*95*	*(79*.*43–85*.*98)*	*10*.*14*	*(10*.*00–10*.*29)*
*Ecstasy*	*1*.*82*	*(1*.*60–2*.*06)*	*70*.*41*	*(63*.*78–76*.*27)*	*6*.*34*	*(6*.*22–6*.*4)*
*Methamphetamine*	*0*.*67*	*(0*.*45–1*.*00)*	*8*.*01*	*(5*.*27–12*.*00)*	*1*.*98*	*(1*.*90–2*.*07)*
*GHB*	*3*.*59*	*(2*.*73–4*.*70)*	*13*.*14*	*(10*.*05–17*.*00)*	*0*.*59*	*(0*.*55–0*.*62)*
*PCP*	*1*.*51*	*(1*.*13–2*.*01)*	*24*.*64*	*(19*.*28–30*.*93)*	*2*.*68*	*(2*.*58–2*.*79)*

### 3.3 Lifetime prevalence of NPS use and sociodemographic characteristics of users

According to our results (Table 1), NPS users were mostly white Americans (84.54%; 95% CI = 77.75%-89.54%), men (77.34%; 95% CI = 71.94%- 81.96%), living on their own (77.21%; 95% CI = 70.27%-82.93%) in large metropolitan areas (54.65%; 95% CI = 48.14%-61.01%). Most NPS users had college level of education of higher (68.49%; 95% CI = 64.01%-72.65%).

The large majority of NPS users were active workers: 49.94% of them work on a full-time basis (95% CI = 44.37%-55.51%) and 25.29% had a part-time job (95% CI = 19.94%-31.50%). As NPS use still was a rare phenomenon during the period under study, comparisons with non-users of NPS and general population appear to be equivalent.

The proportion of people having a household annual income lower than $20,000 was slightly higher among NPS users (32.4%; 95% CI = 26.83%-38.52%) than among non-users (18.29%; 95% CI = 17.98%-18.61%). Similarly, the proportions of people without health insurance (21.23%; 95% CI = 16.64%-26.68%) and receiving government assistance (20.73%; 95% CI = 16.64%-25.52%) were relatively higher among NPS users as compared to non-users (15.43%; 95% CI = 15.21%-15.64% and 17.84%; 95% CI = 17.60%-18.09%, respectively). Consequently, the proportion of people showing no factors of socioeconomic vulnerability as defined by our vulnerability index (cf. 2.3) was lower among NPS users (37.48%; 95% CI = 31.41%-43.97%) than among non-users (45.51%; 95% CI = 45.16%-45.86%). Finally, the use of NPS was more common among young adults under 35 years old, particularly among those aged 18 to 25 that represent about 4 in 10 NPS users. Together, young adults aged 18 to 34 represented 63.76% of NPS users, while they only represented 30.45% of non-users (Table 1). In addition, the lifetime prevalence rates of NPS use for those age groups– 0.49% (95% CI = 0.43%-0.55%) and 0.22% (95% CI = 0.17%-0.28%) respectively–were higher than the overall prevalence rate of 0.17% (95% CI = 0.15%-0.19%) (Table 1).

NPS users with the highest level of education were twice more likely to use an NPS (adjusted OR = 2.46). Conversely, the use of NPS was significantly less likely among people aged between 35–49 (adjusted OR = 0.14; 95% CI = 0.10–0.21) and among African Americans (adjusted OR = 0.32; 95% CI = 0.14–0.581) (Table 2).

**Table 2:** Unadjusted and adjusted binary logistic regression of factors associated with NPS use among adults in the USA, 2007–14.

	Unadjusted			Adjusted ^(a)^		
	OR	95% CI	*p*-Value	OR	95% CI	*p*-Value
**Age**						
18–25	1			1		
26–34	0.44	0.34–0.58	<0.001	0.34	(0.25–0.46)	<0.001
35–49	0.14	0.10–0.21	<0.001	0.14	(0.10–0.21)	<0.001
50 and older	0.20	0.13–0.29	<0.001	0.33	(0.23–0.49)	<0.001
**Sex**						
Female versus Male	0.27	0.20–0.36	<0.001	0.34	(0.25–0.46)	<0.001
**Marital Status**						
Married versus Single	0.26	0.18–0.37	<0.001	0.54	(0.38–0.77)	0.01
**Ethnicity**						
White	1			1		
African American	0.18	0.09–0.37	<0.001	0.32	(0.14–0.58)	0.002
Hispanic	0.40	0.29–0.57	<0.001	0.56	(0.37–0.74)	0.001
Other	0.64	0.24–1.75	0.382	0.97	(0.34–2.76)	0.950
**Metropolitan Area Size**						
Non-Metropolitan	1					
Small Metropolitan	1.35	0.84–2.18	0.209			
Large Metropolitan	1.28	0.76–2.16	0.343			
**Education**						
Elementary	1			1		
Secondary	6.17	2.14–17.81	0.001	1.29	(0.43–3.82)	0.645
Post-secondary	10.22	3.52–29.70	<0.001	2.46	(0.82–7.36)	0.105
**Employment**						
Full-Time	1					
Part-Time	1.87	1.38–2.53	<0.001			
Not employed	0.73	0.54–0.99	0.041			
**Income**						
Less than 20,000	1					
20,000 to 49,999	0.46	0.33–0.64	<0.001			
50,000 to 74,999	0.49	0.33–0.72	0.001			
More than 75,000	0.47	0.34–0.63	<0.001			
**Receives Government Assistance**						
Yes versus no	1.20	0.92–1.58	0.174			
**Health Insurer**						
Private	1					
Medicare	1.23	0.51–2.99	0.647			
Medicaid	0.86	0.59–1.26	0.434			
Tricare & VA	0.90	0.38–2.11	0.800			
Other	2.50	1.38–4.54	0.003			
Uninsured	1.54	1.16–2.05	0.003			
**Economic Vulnerability**						
No vulnerability	1					
Low (1 attribute)	1.43	1.01–2.03	0.042			
Moderate (2 attributes)	1.37	0.97–1.95	0.074			
High (3 or 4 attributes)	1.30	0.91–1.86	0.143			
**Past-Year Serious Psychological Distress**						
Yes versus no	3.05	2.29–4.07	<0.001	1.14	(0.80–1.63)	0.454
**Past-Year Major Depressive Episode**						
Yes versus no	3.07	2.23–4.24	<0.001	1.37	(0.90–2.0)	0.137
**Past-Year Perceived Unmet Mental Health Need**						
Yes versus no	3.43	2.59–4.54	<0.001	1.29	(0.89–1.86)	0.177
**Past-Year Received Mental Treatment**						
Yes versus no	2.26	1.70–3.01	<0.001	1.31	(0.94–1.81)	0.107
**Lifetime use of traditional illicit drugs**						
Yes versus no	91.93	44.64–189.30	<0.001	7.67	(3.49–16.88)	<0.001
*Cocaine*	*30*.*92*	*24*.*69–38*.*72*	<0.001			* *
*Crack*	*10*.*56*	*8*.*21–13*.*57*	<0.001			* *
*Heroin*	*26*.*10*	*19*.*33–35*.*25*	<0.001			* *
*Cannabis*	*90*.*22*	*44*.*20–184*.*18*	<0.001			* *
**Lifetime use of classic synthetic drugs**						
Yes versus no	74.88	52.72–106.35	<0.001	28.44	(19.36–41.77)	<0.001
*LSD*	*43*.*11*	*34*.*34–54*.*12*	<0.001			* *
*Ecstasy*	*35*.*13*	*26*.*04–47*.*39*	<0.001			* *
*Methamphetamine*	*4*.*30*	*2*.*76–6*.*71*	<0.001			* *
*GHB*	*25*.*55*	*18*.*68–34*.*94*	<0.001			* *
*PCP*	*11*.*87*	*8*.*69–16*.*21*	<0.001			* *

(a) Adjusted binary logistic regression for age group, sex, ethnicity, marital status, education, past-year experience of SPD, MDE, unmet mental health need, access to mental health treatment, and lifetime use of traditional illicit drug and classic synthetic drug

**Fig 4:**
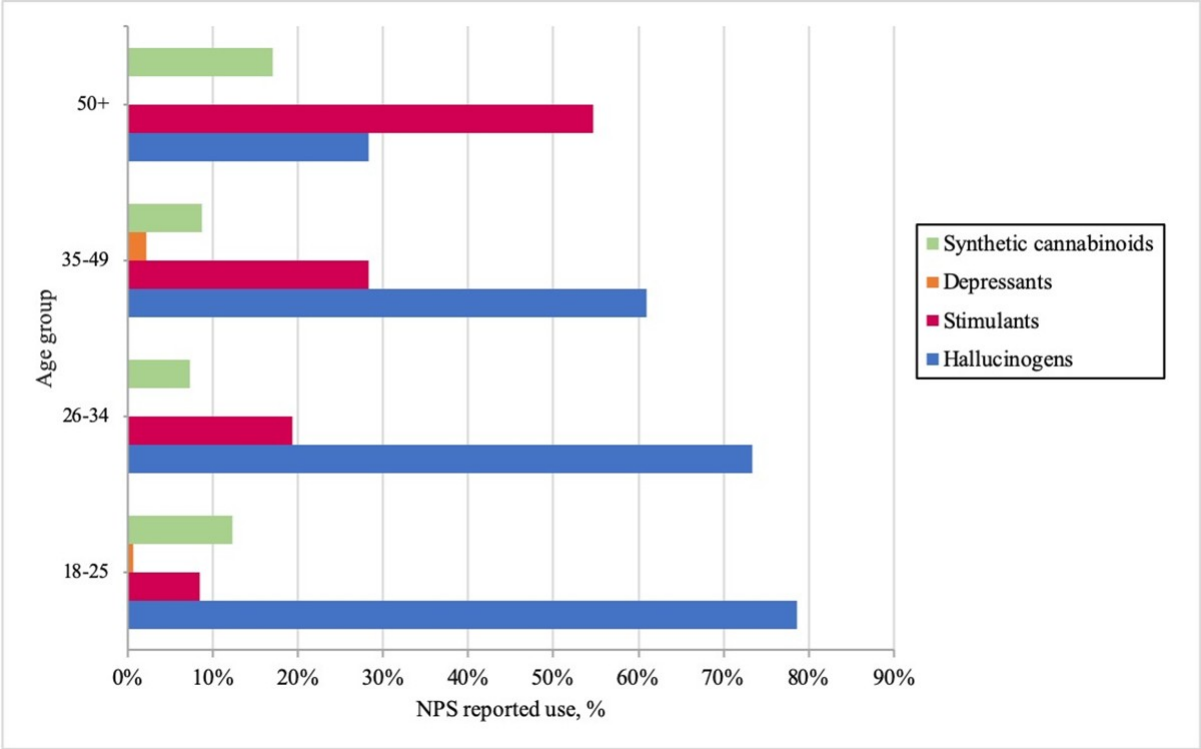
NPS reported use by pharmacological effect and age group, 2007–14.

Different trends in NPS use observed among age groups. The use of hallucinogens was predominantly reported by people aged 34 or younger, especially by young adults under 25 (78.6% of them reported use of NPS hallucinogens). Meanwhile, the use of stimulants seemed to be more common among people aged 50 or older (54.7% of them reported use of NPS stimulants). Interestingly, as illustrated by Fig 4, the use of hallucinogens seems to decrease with age, while the use of stimulants follows the opposite pattern. The use of synthetic cannabinoids was relatively similar across ages.

### 3.4 Patterns of illicit drug use among NPS users

As shown in Table 1, 98.71% (95% CI = 97.38%-99.37%) of NPS users had already consumed at least one of the four most used illicit drugs (e.g. cocaine, crack, heroin and cannabis) in their lifespan. Cannabis and cocaine were the most common illicit drugs consumed by NPS users: almost all of them had already used cannabis (98.66%; 95% CI = 97.31%-99.34%) and 85.46% (95% CI = 82.43%-88.05%) had consumed cocaine at least once in their life (Table 1).

Additionally, 92.43% (95% CI = 89.56%-94.56%) of NPS users had also already used classic synthetic drugs, particularly LSD (82.95%; 95% CI = 79.43%-85.98%) and ecstasy (70.41%; 95% CI = 63.78%-76.27%). The use of PCP (24.64%; 95% CI = 19.28–30.93%), GHB (13.14%; 95% CI = 10.05–17.00%) and methamphetamine (8.01%; 95% CI = 5.27–12.00%) appeared to be less common among NPS users (Table 1). Overall, NPS users seemed to have followed a pattern of polysubstance use throughout their life, which involves both traditional illicit drugs and classic synthetic drugs.

Overall, people having already tried at least one classic synthetic drug in their life were 28.44 times more likely to use an NPS (95% CI = 19.36–41.77, *p* < 0.000) and those that had used traditional illicit drugs were 7.67 times more likely to do so (95% CI = 3.46–16.88, *p* < 0.000) (Table 2).

### 3.5 Use of NPS and mental health

NPS use seemed to be more frequent among adults who had experienced mental health problems. While the overall estimate of lifetime NPS use for adults was 0.17%, it reached 0.42% (95% CI = 0.33%-0.52%) among adults who experienced serious psychological distress (SPD) in the last year and 0.45% (95% CI = 0.34%-0.58%) among those who had a major depressive episode (MDE). Moreover, the estimate for lifetime NPS use was 0.32% (95% CI = 0.25%-0.41%) for adults who received mental health treatment during the last year, while it was 0.51% (95% CI = 0.39%-0.65%) among those that had perceived an unmet mental health need (Table 1).

As shown in Table 2, adults experiencing mental health problems were more exposed to NPS use: adults who had had a MDE over the last 12 months were 1.36 times more likely to have used an NPS (95% CI = 0.90–2.00), while those who experienced SPD in the last year were 1.14 times more likely to have used an NPS (95% CI = 0.80–1.63). Finally, adults with unsolved mental health issues were 1.29 times more likely to have used NPS (95% CI = 0.89–1.86), while those who received mental health treatment were 1.31 times more likely to have used an NPS (95% CI = 0.94–1.81).

## 4. Discussion

According to our findings, the lifetime prevalence of NPS use increased between 2007–14, especially among young adults aged 18 to 25. Although the level of education of NPS users was relatively higher as compared to non-users (or general population), NPS users seemed to have a less wealthy situation. NPS users seemed to have followed a pattern of polysubstance use throughout their life, particularly of cannabis, cocaine and classic synthetic drugs. This is consistent with findings from prior international studies that have already pointed out patterns of polysubstance use among NPS users [11]. As most of the NPS emulate the effects of classic synthetic drugs (e.g. LSD, ecstasy, PCP), their use may be considered as a contributing factor to NPS use. Our study shows that NPS use seems to be more prevalent among people having mental health problems, which is also consistent with prior studies in the field that point to a higher prevalence of NPS use among psychiatric patients, with NPS users being also more likely to require intensive psychiatric care. Moreover, clinical case studies have suggested that the use of synthetic cannabinoids may trigger or aggravate psychotic episodes [44–47]. Considering that scientific evidence has already suggested a bidirectional association between psychotic experiences and selected substance use disorders, the rise in NPS use may have a negative impact on population mental health outcomes [48–50]. As affordability issues are the most frequently mentioned reasons for not receiving mental health services in the USA [51], the association between NPS use and mental health is particularly worrying, especially taking into account the higher proportion of NPS users that declared an unmet mental health need and their relative more precarious socioeconomic situation as compared to non-users. Yet socioeconomic vulnerability appeared to be less important than mental health issues as a correlate to NPS use, as lifetime prevalence of NPS use did not vary according to composite levels of wealth.

The profile of NPS users that may be addressed based on the results of our study (highly educated young adults, economically active yet relatively unprivileged, presenting higher levels of mental health issues than the general population) seem to be concordant with results from recent studies conducted in Europe among NPS users that identify young active workers using NPS to cope with their daily life challenges and dealing with health issues as one of the various profiles of NPS users [52–54].

It is noteworthy to emphasise a few limitations of this study. First, the overall objective of the NSDUH is to provide data on the level and patterns of alcohol, tobacco and illegal substance use and misuse, while the present study particularly aimed to look at the use of NPS based on the data obtained through open-ended questions. In addition, commonly used data collection strategies are known to be inefficient for rare events data such as NPS use, while statistical procedures such as logistic regression, can considerably underestimate the probability of rare events [55]. Hence, most of results obtained through this study are not statistically significant yet they give a valuable input for the study of NPS use as they come from one of the few population-based data sources currently available.

Second, the use of self-reported substance use as an outcome variable may have led to significant levels of mis/underreporting due to the diversity of substances currently available in the drug market (some of whom may have not been reported at all), the variety of nomenclatures associated with them, and the lack of awareness among respondents of the actual chemical composition of the substances they used. Furthermore, the design of the NSDUH as a household survey does not permit sampling of homeless or institutionalized persons, who show specific patterns of problematic drug use [56, 57]. Finally, the use of a repeated, cross-sectional survey provides data about how NPS use has changed over time at a societal level. Unlike a longitudinal study, it does not allow for an assessment of individuals over time and, consequently, relationship cannot be established between the outcome variable and the exposures of interest [58]. Although these findings do not forcibly reflect the current epidemiological correlates of NPS use in the USA, they do provide valuable data on its early stage of development and may inform public health authorities about the main sociodemographic, mental health and drug use patterns of NPS use among adult population.

## 5. Conclusion

The increase in NPS consumption observed over the last decade has raised public health concern about the risks related to the consumption of new substances whose actual origin and composition remained widely unknown. The emergence of NPS is particularly worrying given the scarcity of evidence around the pharmacology and potential toxic effects of new psychoactive substances regularly appearing in the international drug market. Although the coexistence of both problematic drug use and mental health issues is a well-known phenomenon within the scientific community, in the USA this new trend in drug use is of particular interest considering that the proportion of reported unmet mental health need seems to be higher among NPS users as compared to non-users. Further comparative research on trends in NPS use and potential policy public health responses would be instrumental for developing appropriate health interventions, including drug checking, education for users and training for healthcare professionals working both within emergency wards and in/outpatient addiction and mental health services.

## Supporting information

S1 Table Properties and international legal status of NPS reviewed. (DOCX)

## References

[1] U.S. Drug Enforcement Administration. National Forensic Laboratory Information System Special Report: Synthetic Cannabinoids and Synthetic Cathinones Reported in NFLIS, 2010–2013 [Internet]. Springfield; 2014. Available: https://www.nflis.deadiversion.usdoj.gov/DesktopModules/ReportDownloads/Reports/NFLIS_SR_CathCan_508.pdf

[2] Corazza Ornella; Roman-Urrestarazu A. Introduction In: Corazza O, Roman‐Urrestarazu A, editors. Handbook of Novel Psychoactive Substances: What Clinicians Should Know About NPS. New York: Routledge; 2019.

[3] Al‐Imam A, Santacroce R, Roman‐Urrestarazu A, Chilcott R, Bersani G, Martinotti G, et al Captagon: use and trade in the Middle East. Hum Psychopharmacol Clin Exp. Wiley Online Library; 2017;32 10.1002/hup.2548 27766667

[4] Marrinan S, Roman‐Urrestarazu A, Naughton D, Levari E, Collins J, Chilcott R, et al Hair analysis for the detection of drug use—is there potential for evasion? Hum Psychopharmacol Clin Exp. Wiley Online Library; 2017;32 10.1002/hup.2587 28568705

[5] Corazza O, Roman-Urrestarazu A. Editorial Introduction: The Proliferation of NPS as a “Game Changer” for Public Health Policy In: Corazza Ornella; Roman-Urrestarazu A, editor. Novel Psychoactive Substances: Policy, Economics and Drug Regulation. Cham: Springer; 2017.

[6] Wilkins C, Rychert M, Byrska B, Van Hout MC, Corazza O, Roman-Urrestarazu A. Exploring Innovative Policy Responses to NPS and ‘Legal Highs’ in New Zealand, Poland, Republic of Ireland and the UK In: Corazza Ornella; Roman-Urrestarazu A, editor. Novel Psychoactive Substances: Policy, Economics and Drug Regulation. Cham: Springer; 2017 pp. 57–74.

[7] Arnold C. The new danger of synthetic drugs Lancet. Elsevier Ltd; 2013;382: 15–16. 10.1016/s0140-6736(13)61512-3 23841151

[8] United Nations Office on Drugs and Crime. International Standards on Drug Use Prevention [Internet]. Vienna; 2015. Available: https://www.unodc.org/documents/prevention/UNODC_2013_2015_international_standards_on_drug_use_prevention_E.pdf

[9] Corazza O, Chan HY, Roman-Urrestarazu A. NPS: Moving from Blanket Prohibition to a Functionalist Approach In: Corazza Ornella; Roman-Urrestarazu A, editor. Novel Psychoactive Substances: Policy, Economics and Drug Regulation. Cham: Springer; 2017 pp. 125–137.

[10] Tettey JN, Levissianos S. The Global Emergency of NPS: An Analysis of a New Drug Trend In: Corazza Ornella; Roman-Urrestarazu A, editor. Novel Psychoactive Substances: Policy, Economics and Drug Regulation. Cham: Springer; 2017 pp. 1–12.

[11] Soussan C, Kjellgren A. The users of Novel Psychoactive Substances: Online survey about their characteristics, attitudes and motivations. Int J Drug Policy. Elsevier B.V.; 2016;32: 77–84. 10.1016/j.drugpo.2016.03.007 27184218

[12] Schmidt MM, Sharma A, Schifano F, Feinmann C. “Legal highs” on the net-Evaluation of UK-based Websites, products and product information. Forensic Sci Int. 2011;206: 92–97. 10.1016/j.forsciint.2010.06.030 20650576

[13] United Nations Office on Drugs and Crime. World Drug Report 2018—Analysis of Drug Markets: Opiates, cocaine, cannabis, synthetic drugs [Internet]. 2018. Available: https://www.unodc.org/wdr2018/prelaunch/WDR18_Booklet_3_DRUG_MARKETS.pdf

[14] Johnson LA, Johnson RL, Portier R-B. Current “legal highs.” J Emerg Med. 2013;44: 1108–15. 10.1016/j.jemermed.2012.09.147 23528960

[15] United Nations Office on Drugs and Crime. World Drug Report 2020. Cross-cutting issues: evolving trends and new challenges [Internet]. Vienna; 2020. Available: https://wdr.unodc.org/wdr2020/field/WDR20_BOOKLET_4.pdf

[16] European Monitoring Centre for Drugs and Drug Addiction. European Drug Report 2020: Trends and Developments [Internet]. Luxembourg; 2020. Available: https://www.emcdda.europa.eu/system/files/publications/13236/TDAT20001ENN_web.pdf

[17] Baumann MH, Majumdar S, Le Rouzic V, Hunkele A, Uprety R, Huang XP, et al Pharmacological characterization of novel synthetic opioids (NSO) found in the recreational drug marketplace. Neuropharmacology. Elsevier Ltd; 2018;134: 101–107. 10.1016/j.neuropharm.2017.08.016 28807672 PMC5809328

[18] Abdulrahim D, Bowden-Jones O. The misuse of synthetic opioids: harms and clinical management of fentanyl, fentanyl analogues and other novel synthetic opioids. Information for clinicians. London; 2018.

[19] Karila L, Marillier M, Chaumette B, Billieux J, Nicolas F, Amine B. New synthetic opioids: Part of a new addiction landscape. Neurosci Biobehav Rev. 2018; 10.1016/j.neubiorev.2018.06.010 30217656

[20] Armenian P, Vo KT, Barr-Walker J, Lynch KL. Fentanyl, fentanyl analogs and novel synthetic opioids: A comprehensive review. Neuropharmacology. Elsevier Ltd; 2018;134: 121–132. 10.1016/j.neuropharm.2017.10.016 29042317

[21] Lucyk SN, Nelson LS. Novel Synthetic Opioids: An Opioid Epidemic Within an Opioid Epidemic. Ann Emerg Med. American College of Emergency Physicians; 2017;69: 91–93. 10.1016/j.annemergmed.2016.08.445 27745765

[22] Peacock A, Bruno R, Gisev N, Degenhardt L, Hall W, Sedefov R, et al New psychoactive substances: challenges for drug surveillance, control, and public health responses. Lancet. Elsevier Ltd; 2019;394: 1668–1684. 10.1016/S0140-6736(19)32231-7 31668410

[23] Johnston LD, O’Malley PM, Bachman JG, Schulenberg JE. Monitoring the Future. National Results on Drug Use: 2012 Overview Key Findings on Adolescent Drug Use. Institute for Social Research, University of Michigan; 2013.

[24] Palamar JJ, Su MK, Hoffman RS. Characteristics of novel psychoactive substance exposures reported to New York City Poison Center, 2011–2014. Am J Drug Alcohol Abuse. Taylor & Francis; 2016;42: 39–47. 10.3109/00952990.2015.1106551 26678258 PMC4767576

[25] Ma R, Perera S. Safer ‘chemsex’: GPs’ role in harm reduction for emerging forms of recreational drug use. Br J Gen Pract. British Journal of General Practice; 2016;66: 4–5. 10.3399/bjgp16X683029 26719455 PMC4684018

[26] Race K, Lea T, Murphy D, Pienaar K. The future of drugs: recreational drug use and sexual health among gay and other men who have sex with men. Sex Health. CSIRO; 2017;14: 42–50. 10.1071/SH16080 27712616

[27] Wish ED, Billing AS, Artigiani E. Community Drug Early Warning System: The CDEWS‐2 Replication Study. Washington, DC; 2015.

[28] Winstock AR, Barratt MJ, Ferris JA, Maier LJ. Global Drug Survey 2017: Pills, powders, pleasures, problems (Plenary). The 10th International Conference on Nightlife, Substance Use and Related Health Issues. 2017.

[29] U.S. Drug Enforcement Administration. National Forensic Laboratory Information System: Midyear Report 2014. Revised March 2016. [Internet]. Springfield; 2016. Available: https://www.nflis.deadiversion.usdoj.gov/DesktopModules/ReportDownloads/Reports/NFLIS2014MY.pdf

[30] U.S. Drug Enforcement Administration. National Forensic Laboratory Information System. 2016 Annual Report. Revised April 2018. [Internet]. Springfield; 2018. Available: https://www.nflis.deadiversion.usdoj.gov/DesktopModules/ReportDownloads/Reports/NFLIS2016AR_Rev2018.pdf

[31] National Institute on Drug Abuse. Epidemiologic Trends in Drug Abuse. Proceedings of the Community Epidemiology Work Group. Highlights and Executive Summary. [Internet]. 2014. Available: https://archives.drugabuse.gov/sites/default/files/cewgjune2014.pdf

[32] Dart RC, Bronstein AC, Spyker DA, Cantilena LR, Seifert SA, Heard SE, et al Poisoning in the United States: 2012 Emergency Medicine Report of the National Poison Data System. Ann Emerg Med. Elsevier; 2015;65: 416–422. 10.1016/j.annemergmed.2014.11.001 25523411

[33] Riederer AM, Campleman SL, Carlson RG, Boyer EW, Manini AF, Wax PM, et al Acute Poisonings from Synthetic Cannabinoids—50 US Toxicology Investigators Consortium Registry Sites, 2010–2015. Morb Mortal Wkly Rep. NIH Public Access; 2016;65: 692–695. 10.15585/mmwr.mm6527a2 27413997 PMC4972329

[34] Patrick ME, O’Malley PM, Kloska DD, Schulenberg JE, Johnston LD, Miech RA, et al Novel psychoactive substance use by US adolescents: characteristics associated with use of synthetic cannabinoids and synthetic cathinones. Drug Alcohol Rev. Wiley Online Library; 2016;35: 586–590. 10.1111/dar.12372 26711540 PMC4927404

[35] Fernández-Calderón F, Cleland CM, Palamar JJ. Polysubstance use profiles among electronic dance music party attendees in New York City and their relation to use of new psychoactive substances. Addict Behav. Elsevier; 2018;78: 85–93. 10.1016/j.addbeh.2017.11.004 29128711 PMC5783759

[36] Palamar JJ, Barratt MJ, Ferris JA, Winstock AR. Correlates of New Psychoactive Substance Use Among a Self‐Selected Sample of Nightclub Attendees in the United States. Am J Addict. Wiley Online Library; 2016;25: 400–407. 10.1111/ajad.12403 27419383 PMC5072356

[37] Murphy CM, Dulaney AR, Beuhler MC, Kacinko S. “Bath Salts” and “Plant Food” Products: the Experience of One Regional US Poison Center. J Med Toxicol. Springer; 2013;9: 42–48. 10.1007/s13181-012-0243-1 22733603 PMC3576506

[38] Hunter LJ, Dargan PI, Benzie A, White JA, Wood DM. Recreational drug use in men who have sex with men (MSM) attending UK sexual health services is significantly higher than in non-MSM. Postgrad Med J. The Fellowship of Postgraduate Medicine; 2014;90: 133–138. 10.1136/postgradmedj-2012-131428 24390619

[39] Bourne A, Reid D, Hickson F, Torres-Rueda S, Weatherburn P. Illicit drug use in sexual settings (‘chemsex’) and HIV/STI transmission risk behaviour among gay men in South London: findings from a qualitative study. Sex Transm Infect. BMJ Publishing Group Ltd; 2015;91: 564–568. 10.1136/sextrans-2015-052052 26163510

[40] Center for Behavioral Health Statistics and Quality. 2015 National Survey on Drug Use and Health: Summary of the Effects of the 2015 NSDUH Questionnaire Redesign: Implications for Data Users [Internet]. Rockville, MD; 2016. Available: https://www.samhsa.gov/data/sites/default/files/NSDUH-TrendBreak-2015.pdf 30199192

[41] Center for Behavioral Health Statistics and Quality. 2014 National Survey on Drugs Use and Health. Methodological Summary and Definitions. [Internet]. Rockville, MD; 2015. Available: https://www.samhsa.gov/data/report/2014-national-survey-drug-use-and-health-methodological-summary-and-definitions

[42] Center for Behavioral Health Statistics and Quality. National Survey on Drug Use and Health, 2014. Codebook. [Internet]. Rockville, MD; 2015. 10.1186/1472-6807-9-19

[43] Jann B. Tabulation of multiple responses. Stata J. 2005;5: 92–122. Available: https://www.stata-journal.com/sjpdf.html?articlenum=st0082

[44] Place C, Scally A, Gow L, Wade A, Barrowcliff R, Nasim I, et al Spice boys: an exploratory study around novel psychoactive substance use on a male acute ward. Adv Dual Diagn. 2017;10: 97–104. 10.1108/ADD-10-2016-0018

[45] Every-Palmer S. Synthetic cannabinoid JWH-018 and psychosis: An explorative study. Drug Alcohol Depend. Elsevier Ireland Ltd; 2011;117: 152–157. 10.1016/j.drugalcdep.2011.01.012 21316162

[46] Schifano F, Orsolini L, Duccio Papanti G, Corkery JM. Novel psychoactive substances of interest for psychiatry. World Psychiatry. World Psychiatric Association; 2015;14: 15–26. 10.1002/wps.20174 25655145 PMC4329884

[47] United Nations Office on Drugs and Crime. World Drug Report 2017. Market Analysis of Synthetic Drugs: Amphetamine-type stimulants, new psychoactive substances [Internet]. UNODC Vienna; 2017 Available: https://www.unodc.org/wdr2017/field/Booklet_4_ATSNPS.pdf

[48] Degenhardt L, Saha S, Lim CCW, Aguilar-Gaxiola S, Al-Hamzawi A, Alonso J, et al The associations between psychotic experiences and substance use and substance use disorders: findings from the World Health Organization World Mental Health surveys. Addiction. 2018;113: 924–934. 10.1111/add.14145 29284197 PMC5895500

[49] Simonato P, Corazza O, Santonastaso P, Corkery J, Deluca P, Davey Z, et al Novel psychoactive substances as a novel challenge for health professionals: results from an Italian survey. Hum Psychopharmacol. 2013;28: 324–331. 10.1002/hup.2300 23881880

[50] Martinotti G, Merino del Villar C, Giorgetti R, Schifano F, Di Giannantonio M. Novel and Traditional Club Substances’ Association to Psychopathological and Medical Sequelae. The Ibiza Project In: Corazza O, Roman-Urrestarazu A, editors. Handbook of Novel Psychoactive Substances: What Clinicians Should Know About NPS. New York: Routledge; 2019.

[51] Center for Behavioral Health Statistics and Quality. The NSDUH Report Data Spotlight: Data Spotlight [Internet]. Rockville, MD; 2013. Available: http://www.samhsa.gov/data/spotlight/spot075-services-affordability-2013.pdf

[52] Benschop A, Urbán R, Kapitány-Fövény M, Van Hout MC, Dąbrowska K, Felvinczi K, et al Why do people use new psychoactive substances? Development of a new measurement tool in six European countries. J Psychopharmacol. 2020;34: 600–611. 10.1177/0269881120904951 32043399

[53] Simonis S, Canfyn M, Van Dijck A, Van Havere T, Deconinck E, Blanckaert P, et al Awareness of users and motivational factors for using new psychoactive substances in Belgium. Harm Reduct J. Harm Reduction Journal; 2020;17: 1–11. 10.1186/s12954-019-0355-x 32711526 PMC7382100

[54] Soussan C, Andersson M, Kjellgren A. The diverse reasons for using Novel Psychoactive Substances—A qualitative study of the users’ own perspectives. Int J Drug Policy. Elsevier B.V.; 2018;52: 71–78. 10.1016/j.drugpo.2017.11.003 29241144

[55] King G, Zeng L. Logistic Regression in Rare Events Data. J Stat Softw. 2003;8.

[56] Carter Narendorf S, Cross MB, Santa Maria D, Swank PR, Bordnick PS. Relations between mental health diagnoses, mental health treatment, and substance use in homeless youth. Drug Alcohol Depend. Elsevier Ireland Ltd; 2017;175: 1–8. 10.1016/j.drugalcdep.2017.01.028 28364629

[57] Yang JC, Roman-Urrestarazu A, Brayne C. Binge alcohol and substance use across birth cohorts and the global financial crisis in the United States. PLoS One. 2018;13: 1–18. 10.1371/journal.pone.0199741 29940033 PMC6016915

[58] Merikangas KR, McClair VL. Epidemiology of substance use disorders. Hum Genet. 2012;131: 779–789. 10.1007/s00439-012-1168-0 22543841 PMC4408274

