# Correction: Lifetime prevalence of novel psychoactive substances use among adults in the USA: Sociodemographic, mental health and illicit drug use correlates. Evidence from a population-based survey 2007–2014

**DOI:** 10.1371/journal.pone.0251006

**Published:** 2021-04-27

**Authors:** Jessica Neicun, Justin Christopher Yang, Hueyjong Shih, Pranay Nadella, Robin van Kessel, Attilio Negri, Katarzyna Czabanowska, Carol Brayne, Andres Roman-Urrestarazu

The seventh author’s name is spelled incorrectly. The correct name is: Katarzyna Czabanowska.
