# Supplementary material for: Lifetime prevalence of novel psychoactive substances use among adults in the USA: Sociodemographic, mental health and illicit drug use correlates. Evidence from a population-based survey 2007–2014
Source: PLoS One. 2020 Oct 30;15(10):e0241056. doi: 10.1371/journal.pone.0241056 (PMC7598490; doi:10.1371/journal.pone.0241056)
Supplement: S1 Table — (DOCX) [file pone.0241056.s001.docx]

**S1 Table.** Properties and international legal status of NPS reviewed

| **Drug class** | **Drug name** | **Chemical name (IUPAC name)** | **International legal status *** |
| --- | --- | --- | --- |
| **Hallucinogens** |  |  |  |
| Phenethylamines | 2C-B (Bromo mescaline, Nexus) | 4-Bromo-2,5-dimethoxyphenethylamine | UN Schedule II ^a^ |
|  | 25I-NBOMe (2C-I-NBOMe, BOM-CI, Cimbi-5) | 2-(4-Iodo-2,5-dimethoxyphenyl)-N-[(2-methoxyphenyl)methyl]ethanamine | UN Schedule I ^b^ |
|  | 25B-NBOMe (2C-B-NBOMe, 25B, Cimbi-36) | [2-(4-bromo-2,5-dimethoxyphenyl)ethyl][(2-methoxyphenyl)methyl]amine | UN Schedule I ^b^ |
|  | 25C-NBOMe (2C-C-NBOMe, 25C, N-Bomb, Cimbi-82) | 2-(4-Chloro-2,5-dimethoxyphenyl)-N-[(2-methoxyphenyl)methyl]ethanamine | UN Schedule I ^b^ |
|  | 2C-T-7 (T7, "Blue mystic") | 2-[2,5-Dimethoxy-4-(propylthio)phenyl]ethanamine |  |
|  | 2C-T-2 | 2-[4-(Ethylthio)-2,5-dimethoxyphenyl]ethanamine |  |
|  | 2C-I | 2,5-Dimethoxy-4-iodophenethylamine |  |
|  | 2C-T-21 | 2,5-Dimethoxy-4-(2-fluoroethylthio)phenethylamine |  |
|  | 2C-E | 2-5-Dimethoxy-4-ethyl-phenethylamine |  |
|  | 2C-C | 2,5-Dimethoxy-4-chlorophenethylamine |  |
|  | 2C-X |  |  |
|  | 2C-D | 2,5-Dimethoxy-4-methylphenethylamine |  |
|  | 2C-T | 2,5-Dimethoxy-4-methylthiophenethylamine |  |
|  | 2C-BCB-NBOMe (2-TCB-NBOMe) |  |  |
|  | 2C-P | 4-propyl-2,5-dimethoxyphenethylamine |  |
|  | 2C-F | 2,5-Dimethoxy-4-fluorobenzaldehyde |  |
|  | DOA | 2,5-Dimethoxyamphetamine; 2,5-DMA |  |
|  | DOI | 2,5-Dimethoxy-4-iodoamphetamine |  |
|  | DOC | 2,5-dimethoxy-4-chloroamphetamine |  |
|  | DOM/STP | 2,5-Dimethoxy-4-methylamphetamine | UN Schedule I ^a^ |
|  | 3C-Bromo-Dragonfly (DOB-Dragonfly) | 1-(4-Bromofuro[2,3-f] [1]benzofuran-8-yl)propan-2-amine |  |
|  | Brolamfetamine (DOB) | 2,5-dimethoxy-4-bromoamphetamine | UN Schedule I ^a^ |
| * Source: Schedules of the Convention on Psychotropic Substances of 1971, as at 19 November 2019 | | | |
| ^a^ Substances placed under international control, recently made available and/or used for recreational purposes | | | |
| ^b^ Substances placed under international control after the collection of the survey data | | | |

| **Drug class** | **Drug name** | **Chemical name (IUPAC name)** | **International legal status *** |
| --- | --- | --- | --- |
| **Hallucinogens** |  |  |  |
| Tryptamines | 5-MeO-DMT | 5-methoxy-N,N-dimethyltryptamine |  |
|  | 5-MeO-DiPT ("Foxy methoxy") | 5-Methoxy-N,N-diisopropyltryptamine |  |
|  | 5-MeO-AMT (α,O-Dimethylserotonin, Alpha-O) | 5-methoxy-α-methyltryptamine |  |
|  | 5-MeO-DALT | N,N-diallyl-5-methoxytryptamine |  |
|  | 5-MeO-MIPT | 5,6-dimethoxy-N-methyl-N-isopropyltryptamine |  |
|  | 5-MeO (otherwise unspecified) |  |  |
|  | 4-MeO-DMT | 4-methoxy-N,N-dimethyltryptamine |  |
|  | 4-AcO-DMT | 4-acetoxy-N,N-dimethyltryptamine |  |
|  | 4-HO-DiPT (Iprocin) | 4-hydroxy-DMT |  |
|  | 4-AcO-DiPT (Ipracetin) | 4-Acetoxy-DiPT |  |
|  | 4-HO-MiPT (Miprocin) | 4-Hydroxy-MIPT |  |
|  | 4-HO-MET (Metocin) | 4-hydroxy-N-methyl-N-ethyltryptamine |  |
|  | Methylisopropyltryptamine (MIPT) | N-Methyl-Nisopropyltryptamine |  |
|  | Dipropyltryptamine (DPT, "The Light") | N,N-Dipropyltryptamine |  |
|  | Diisopropyltryptamine (DiPT) | N,N-diisopropyltryptamine |  |
|  | Alpha‐methyltryptamine (AMT, IT-290, 3-IT, αMT) | 1-(1H-Indol-3-yl)propan-2-amine |  |
|  | Trimethoxyamphetamine (TMA) | (±)-3,4,5-Trimethoxy-α-methylphenethylamine | UN Schedule I ^a^ |
| Other hallucinogens | ALD-52 | 1-Acetyl-N,N-diethyllysergamide |  |
|  | Lysergic acid (LSZ) | 2,4-dimethylazetidide |  |
| **Stimulants** |  |  |  |
| Synthetic cathinones | Mephedrone; 4-methylmethcathinone; MCAT | (RS)-2-(Methylamino)-1-(4-methylphenyl)propan-1-one | UN Schedule II ^b^ |
|  | Methedrone; 4-methoxymethcathinone; βk-PMMA | - (RS)-1-(4-Methoxyphenyl)-2-(methylamino)propan-1-one |  |
|  | 3,4-Methylenedioxypyrovalerone (MDPV) | (RS)-1-(1,3-benzodioxol-5-yl)-2-(pyrrolidin-1-yl)pentan-1-one | UN Schedule II ^b^ |
|  | Methylone (MDMC, βk-MDMA) | (RS)-2-Methylamino-1-(3,4-methylenedioxyphenyl)propan-1-one | UN Schedule II ^b^ |
| * Source: Schedules of the Convention on Psychotropic Substances of 1971, as at 19 November 2019 | | | |
| ^a^ Substances placed under international control, recently made available and/or used for recreational purposes | | | |
| ^b^ Substances placed under international control after the collection of the survey data | | | |
| **Drug class** | **Drug name** | **Chemical name (IUPAC name)** | **International legal status *** |
| **Stimulants** |  |  |  |
| Synthetic cathinones | Synthetic cathinones / street names (e.g.: Bath salts, Cloud 9, Cloud 13) |  |  |
| Phenethylamines | Tenamfetamine (MDA) | α-Methyl-3,4-(methylenedioxy)phenethylamine | UN Schedule I ^a^ |
|  | Paramethoxyamphetamine (PMA) | p-Methoxy-α-methylphenylethylamine | UN Schedule I ^a^ |
|  | N-Ethyl MDA (MDE, "Eve") | (±)-N-Ethyl-α-methyl-3,4-(methylenedioxy)phenethylamine | UN Schedule I ^a^ |
|  | Para-Methoxymethylamphetamine (PMMA) | 1-(4-Methoxyphenyl)- 2-aminopropane | UN Schedule I ^a^ |
|  | EDMA | 3,4-Ethylenedioxy-N-methylamphetamine |  |
|  | 4-FMA | (RS)-1-(4-Fluorophenyl)-N-methylpropan-2-amine |  |
|  | 6-APB (Benzo Fury) | 6-(2-aminopropyl)benzofuran |  |
| Other stimulants | Benzylpiperazine (BZP) | 1-Benzylpiperazine | UN Schedule II ^a^ |
|  | MDAI | 5,6-methylenedioxy-2-aminoindane |  |
| **Depressants** |  |  |  |
| Benzodiazepines | Etizolam | 4-(2-chlorophenyl)-2-ethyl-9-methyl-6H-thieno[3,2- f][1,2,4]triazolo[4,3-a][1,4]diazepine |  |
|  | Phenezepam | 7-Bromo-5-(2-chlorophenyl)-1,3-dihydro-2H-1,4-benzodiazepin-2-one |  |
| Opioids | Synthetic heroin (otherwise unspecified) |  |  |
| Dissociatives | GBL | γ-Butyrolactone |  |
|  | GBL / street names (e.g.: Herbal ecstasy, Green hornet, Liquid ecstasy) |  |  |
| **Synthetic cannabinoids** | AM-2201 | (1-(5-Fluoropentyl)-1H-indol-3-yl1-naphthyl)methanone | UN Schedule II ^b^ |
|  | CP55,940 |  |  |
|  | Synthetic cannabinoids / street names (e.g.: K2, Spice, JWH-018, Binaca, Purple haze, Zombie matter, Cloud 10 Ultra, K4, 7H) |  |  |
| * Source: Schedules of the Convention on Psychotropic Substances of 1971, as at 19 November 2019 | | | |
| ^a^ Substances placed under international control, recently made available and/or used for recreational purposes | | | |
| ^b^ Substances placed under international control after the collection of the survey data | | | |
